# Motivational Interviewing as an intervention to increase adolescent self-efficacy and promote weight loss: Methodology and design

**DOI:** 10.1186/1471-2458-11-459

**Published:** 2011-06-10

**Authors:** Beverly Walpole, Elizabeth Dettmer, Barbara Morrongiello, Brian McCrindle, Jill Hamilton

**Affiliations:** 1 Department of Psychology, University of Guelph, Guelph, Canada; 2 Hospital for Sick Children, Toronto, Canada; 3 University of Guelph, Guelph, Canada; 4 Division of Cardiology, Hospital for Sick Children, Toronto, Canada; 5 Division of Endocrinology, Hospital for Sick Children, Toronto, Canada

## Abstract

**Background:**

Childhood obesity is associated with serious physiological and psychological consequences including type 2 diabetes, higher rates of depression and low self-esteem. With the population of overweight and obese youth increasing, appropriate interventions are needed that speak to the issue of readiness to change and motivation to maintain adherence to healthy behavior changes. Motivational Interviewing (MI) is a method of therapy found to resolve ambivalence, enhance intrinsic motivation and promote confidence in a person's ability to make behavior changes. While MI has shown promise in the adult obesity literature as effecting positive lifestyle change, little is known about the effectiveness of MI with overweight and obese youth. This study aims to: 1) demonstrate that MI is an effective intervention for increasing a person's self-efficacy; 2) demonstrate that exposure to MI will facilitate healthy behavior changes; 3) explore psychological changes related to participation in MI and 4) compare physiological and anthropometric outcomes before and after intervention.

**Methods/Design:**

The current investigation is a prospective study conducted with ongoing participants who regularly attend an outpatient pediatric care center for weight-loss. Overweight youth (BMI > 85^th ^%ile) between the ages of 10 and 18 who meet eligibility criteria will be recruited. Participants will be randomly assigned to a control group (social skills training) or a treatment group (MI). Participants will meet with the therapist for approximately 30 minutes prior to seeing the dietician, over the course of 6 months. Participants will also undergo a full day assessment at the beginning and end of psychology intervention to evaluate body fat, and metabolic risk (screening for diabetes, high cholesterol, high blood pressure and fitness level). The paper and pencil portions of the assessments as well as the clinical testing will occur at baseline and at the conclusion of the intervention (6 months) with a repeat assessment 6 months following the completion of the intervention.

**Discussion:**

Results from this study are expected to enhance our understanding of the efficacy of MI with children and adolescents who are overweight or obese.

**Trial registration:**

Current Controlled Trials #NCT00326404.

## Background

The prevalence of overweight and obesity has risen dramatically among North American children and adolescents over the past 30 years [1]. Although the etiology of obesity remains a topic of great debate, evidence clearly shows that obesity is associated with serious, physiological and psychological consequences. Physical consequences related to obesity include type 2 diabetes, hypertension, hyperlipidemia, insulin resistance, asthma, sleep-apnea, and increased likelihood of mortality [2,3]. Moreover, it has be shown that by age 10, 60% of overweight children have at least one biochemical or clinical cardiovascular risk factor and 25% have more than two [4]. Even more alarming are future projections which indicate that if current trends continue, one third of the children born in the year 2000 will eventually develop type 2 diabetes [5]. In addition to the physiological outcomes associated with being overweight or obese, these children are also likely to experience severe psychological and emotional problems as a result of the harmful social stigma and ridicule. Specifically, research has shown that these children are stereotyped as unhealthy, academically unsuccessful, socially inept, unhygienic, and lazy [6]. Furthermore, overweight and obese youth are more likely to be the victims of verbal, physical, and relational bullying, and they experience more teasing and peer rejection than do their normal-weight peers [7]. The aforementioned social problems are predictive of both short and long-term psychological consequences, including: body dissatisfaction, low self-esteem, poorer health-related quality of life and higher rates of depression [8,9].

### Traditional Psychological Interventions for Weight-Loss

Improving childhood weight status and health outcomes will require concerted effort at multiple levels of intervention and counseling by health care professionals represents an important component of the public health response [10]. Cognitive-behavioral treatment has been found to effect positive behavior changes for weight-loss [11]. This psychological method of treatment is based on the theory that the problem in question (eg, obesity) is maintained by specific dysfunctional cognitions and beliefs. The CBT therapist therefore attempts to modify behaviors using techniques designed to identify, evaluate, and then restructure related maladaptive cognitions and beliefs [11].

Despite the benefits of CBT, there are limitations to this approach, which have implications for a client's adherence to a prescribed health behavior program. For instance, CBT can be a direct approach whereby clients' beliefs are challenged and they are directed to explore the evidence that counters their present schema. This method may therefore not be as effective if clients are not ready to recognize maladaptive belief patterns (i.e., they are in a pre-contemplative stage of change). As well, CBT is not designed to engender intrinsic motivation to make necessary changes, again which can have implications for program adherence. Adherence to a weight-loss treatment protocol, such as consuming the recommended amount of calories, becomes a critical component of success. In fact, continued adherence to the prescribed diet has been found to be essential to weight loss success, regardless of the type of diet [12]. Taken together, research suggests that CBT shows a mixture of results [13] and can fall short in some important areas. Thus, alternate treatments of obesity are needed, and particular attention needs to be paid to interventions that speak to the issue of readiness to change and motivation to maintain program adherence.

### Motivational Interviewing as an Intervention for Weight-Loss

One empirically supported intervention with a large evidentiary base for improving adult outcomes in behavioral health-related disorders is Motivational Interviewing (MI). MI is strongly rooted in the client-centered therapy of Rogers [14]. Its relational stance emphasizes the importance of understanding the client's internal frame of reference and displaying unconditional positive regard for the client. MI can thus be defined as a client-centered, directive method of therapy for enhancing intrinsic motivation to change by exploring and resolving ambivalence [15]. MI manifests through specific strategies, such as reflective listening, summarization, shared decision making, and agenda setting. A notable feature of this approach is the importance of the therapist's empathic attunement to emergent client concerns and the communication of accurate understanding. The therapist's capacity to embody the MI spirit and establish a secure empathic bond with a client is thought to be central towards achieving positive treatment outcomes [16].

In addition to empathic responsivity, MI also has a directive component which specifies goals of reducing ambivalence and increasing a client's own intrinsic motivation to change without countering an individual's previously held beliefs. In fact, an MI therapist typically makes no direct attempt to confront denial, dismantal irrational or maladaptive beliefs, or persuade. Confrontation with a client's personal beliefs can damage the therapeutic rapport and build defensiveness rather than confidence. Instead, the therapist helps the client think about and verbally express their own reasons for and against change and how their current behavior or health status affects their ability to achieve their life goals or fulfill core values [17]. Thus MI has proven to be a valuable tool for eliciting intrinsic motivation to make changes by encouraging clients to find their own means and their own solutions [18].

### Why does MI Work? Intrinsic Factors Related to Behavior Change

When attempting to explain the intrinsic factors essential to MI, it is helpful to first consider a theoretical model of behavior change. Prochaska's transtheoretical model of change, for instance, breaks down the concept of readiness to change into stages from pre-contemplation (i.e., not at all contemplating change) to contemplation, action and maintenance of the behavior change once it is made [19]. This model emphasizes the progression of behavior change through stages on a temporal dimension. That individuals are not uniformly in one stage of change, but rather tend to move back and forth fluidly between stages, is an important consideration of the behavior change model [20].

Motivational interviewing is an evocative technique that may be used to facilitate progression through the stages of change. It is based on the assumption that an individual's readiness to change is variable and dynamic, and that different therapeutic techniques will be more constructive depending on their readiness for change. MI is thought to be particularly useful for people who are in the early stages of change as it is in these stages that individuals' consciousness about particular behavior is raised. Over time, MI helps individuals move from being unaware or unwilling to do anything about the problem to considering the possibility of change, to becoming determined and prepared to make the change [21]. Furthermore, most behavior change models include the idea that there are at least two driving components to readiness to change: conviction/importance and confidence/self-efficacy [15]. 'Importance' relates to why change is needed or what is driving the individual's desire to make a change, while 'confidence,' relates to the person's belief in their own ability to master change. Motivational Interviewing works on *both *of these dimensions by helping the client to articulate why it is important for them to change and by increasing self-efficacy so that they have confidence to do so [21].

### Previous MI Intervention Studies

According to Rollnick, Miller and Butler [22], support for the process of change as facilitated by MI has been demonstrated in many studies focusing on adult behavior changes, including drug and alcohol addiction, gambling and adherence to exercise and dietary modification. As such, while the behavior target can differ, the structure of the change process appears to be the same and the fundamental spirit of MI is revered. Using MI to effect weight-loss has been gaining more attention in recent years [22]. Research has demonstrated that MI, in conjunction with standard treatment/treatment as usual (diet and exercise), has resulted in increased success (increased weight loss) for clients enrolled in adult weight loss programs [23]. Moreover, studies have found that adults who show increases in self-efficacy are more likely to adhere to dietary programs and are thus more inclined to lose weight [24]. However, the process by which MI effects change is lacking a model to explain the driving behavior mechanisms. As a result, the relationship between MI and self-efficacy is not well understood, particularly with youth.

Using Motivational Interviewing with children as a therapeutic technique for effecting behavior change is in the beginning stages [17]. While there is some support for MI in adherence-related interventions involving youth (e.g., diabetes, asthma), the evidence base for the use of MI for overweight and obese youth remains limited [25]. Two studies examined the effectiveness of using MI to effect changes in BMI. The "Go Girls" project [26] divided adolescent girls ages 12-16 into high intensity (>20 behavioral intervention sessions) or a moderate intensity (≈6 sessions over the telephone) groups. No significant differences were found between groups at 6-month follow-up. The second study that was described, the "Healthy Lifestyles Pilot Study" looked at using MI with parents of overweight children ages 3-7. Parents in the low-intervention treatment group received one single session of MI, while the high-intensity group received two to four MI sessions [27]. While decreases in BMI from baseline scores were demonstrated, significant differences between groups were not found.

The aforementioned MI studies raise many questions related to the methodological reliability and efficacy of using this therapeutic technique with pediatric samples. We will aim to address these knowledge gaps within the context of our ongoing cohort of obese and overweight youth. The specific purpose of this report is to present the methodology and design of the study investigating the efficacy of Motivational Interviewing on the impact of psychological and physiological wellbeing.

## Methods/Design

This interventional study is part of a broader research program at the Hospital for Sick Children and is funded by the Canadian Institute for Health and Research (CIHR), "HISTORY: High Impact Strategies Towards Overweight Reduction in Youth: Obesity Team Grant." Cooperation from clinics from which subjects will be recruited has already been established, as have the relevant testing protocols. This registered clinical trial (## NCT00326404) is currently being carried out with the ethical approval of the Hospital for Sick Children (REB# 100001762), Toronto East General Hospital (REB# 427-1008-Ped-016) and the University of Guelph (REB# 10JL030).

### Participants

Overweight and obese youth, with BMI >85^th ^%ile for age and gender (as classified by the Center for Disease Control) [28], age 10-18 years, attending a local pediatric outpatient obesity clinic (Healthy Lifestyles Clinic) will be eligible to participate. Time of entry into the weight-loss program will also be controlled for; however, both new and current participants will have the option to participate in the study. Individuals will be excluded if they: are taking medication whose side effects may influence weight gain or weight loss, do not speak English, have known developmental delay, report being pregnant and/or report having an active eating disorder. Verbal and written consent/assent will be obtained from adolescents (and parents if under 16) at the treatment site.

### Procedures/Interventions

#### Assessment and testing at the Hospital for Sick Children

Prior to receiving intervention, all participants will be asked to attend a half-day medical assessment at the Hospital for Sick Children in Toronto at the beginning and end of the study (6 months after their baseline testing). This testing will be carried out in the clinical investigation unit (CIU) and the exercise laboratory, by trained personnel. Clients will undergo a series of physiological tests in order to assess their metabolic health and physical functioning. Specific elements to be measured include: body composition (body fat percentage), body mass index calculation (weight[kg]/height[m^2^], waist circumference, metabolic profile (including oral glucose tolerance test to screen for diabetes, fasting lipid profile, hepatic enzyme profile [to screen for nonalcoholic fatty liver disease], measurement of various adipokines known to be associated with obesity [leptin, adiponectin, highly sensitive c-reactive protein], blood pressure assessment), fitness level (calculated as VO2 max with testing on a cycle ergometer) and nutritional assessment (using a pediatric-validated food frequency questionnaire) ***(See Appendix A for detailed assessment plan)***. All of these measures have been performed routinely as part of the HISTORY "Determinants of metabolic risk" study. This protocol is performed on a single day, with participants arriving at 8 am and finishing by 2 pm. In the event that subjects decline this medical testing, they will not be excluded from participating in the MI part of the study.

#### On-site therapy intervention

Subjects will be recruited directly by the primary investigators from the Toronto East General Hospital's Healthy Lifestyles program. This program is comprised of children and adolescents ages 10-18, who are seeking diet and exercise treatment for their obesity. The Healthy Lifestyles clinic runs weekly on Fridays and all participants are seen for assessment by a pediatric physician as well as by the registered pediatric dietitian. On average between 5 and 8 clients are seen each week. The current standard treatment program includes monthly visits with regular dietary and activity counseling performed by the dietician. There is no psychologist or mental health worker affiliated with the program at this time. This program is comprised of children and adolescents ages 3-18.

A research investigator will recruit youth on site, by introducing the study and inviting their participation. All participants from the secondary care program will be considered, however, participation in the study will be completely optional.

Participants who consent to receiving therapy in addition to their usual care will be randomly assigned to one of two therapies: Motivational Interviewing (experimental arm) or social skills training (control arm). Social skills training will be conducted using a standardized treatment manual, appropriate for this age range. Sessions are based around finding appropriate ways to navigate typical social situations (e.g., how to negotiate with parents). For the MI treatment group, a clinical psychology doctoral student trained in Motivational Interviewing will administer approximately six individual motivational interviewing treatment sessions approximately 30 minutes in length. Sessions will be tape recorded and a percentage (approximately 25%) coded by coders trained in the Motivational Interviewing Treatment Integrity Code (MITI). A smaller percentage (approximately 5%) of the control group sessions will also be randomly checked by a trained MINT trainer to ensure that MI is not being conducted with the control group. While the MI literature indicates that certain methodological parameters (e.g., optimal number of treatment sessions) have not been established, studies have shown that six MI sessions can effect significant behavioral changes in adolescents [29]. The current MI treatment will take place at the Toronto East General Hospital, in a private room. To assess the validity of treatment implementation, experts trained in MI outside of the study, will independently and randomly review and rate approximately 25% of recorded therapy sessions. This will serve as a manipulation check.

The control group will receive six sessions of social skills training. Sessions will be matched for session frequency and duration. This administrator will not be trained in motivational interviewing. These sessions will also be audio taped and reviewed by an MI expert to verify that MI techniques have not been used.

All therapy sessions will take place over the course of 6 months, and will occur at the time of the client's regular follow-up visits to the treatment site (≈ once per month). Therefore, clients will not be required to attend sessions in addition to their regularly scheduled appointments. Consenting participants will spend their usual time with the dietitian as per regular follow-up protocol, and an additional 30 minutes with the therapy provider.

In addition to the monthly therapy sessions, participants will receive monthly telephone check-ins. These "booster sessions" are expected to be approximately 5 minutes, and will be conducted with both control and treatment groups as a means of increasing program adherence and strengthening the therapeutic alliance.

Questionnaires ***(See ***Table 1*** for a list of measures) ***will be administered at the beginning of the study, immediately after intervention (6 months from baseline) and 12 months after baseline. Measures used in this study have been validated for use in child and adolescent age groups

**Table 1:** Table of Measures

Questionnaire	Age Group used for Validation	Number of Items	Length of Time Estimated to Complete (minutes)
Readiness to Change	9-adult	4	1

Dutch Eating Behavior Questionnaire (DEBQ)	9-adult	20	6

Rosenberg Self-Esteem Scale	School-age children- adults	10	3

Children's Depression Inventory-S (CDI)	School-age children- adolescents	10	3

Weight-Loss Behavior Scale	Adolescents	36	8

Child Dietary Self-Efficacy Scale (CDSS)	School-age children	10	3

Adolescent Coping Questionnaire (A-COPE)	Adolescents	52	15

PedsQL™	8-13	20	6

Difficulties in Emotion Regulation Scale-emotional eating scale (DERS)	Adolescent-Adult	13 items in emotional eating scale	5

Weight Efficacy Lifestyle questionnaire	Adults	20	6

HAES exercise Scale	Child/Adolescent	20	6

Demographics/Clinical Report Form (CRF)	Parent of Child/Adult	12	4

**TOTAL**		**190**	**60**

#### Study Objectives

The present study is designed to explore the impact of Motivational Interviewing with a sample of overweight and obese youth. The main specific objectives are as follows:

1. To demonstrate that Motivational Interviewing is an effective intervention for increasing a person's belief in their ability (i.e., self-efficacy) to adhere to healthy behavior changes.

2. To demonstrate that exposure to Motivational Interviewing will facilitate healthy behavior changes (increased activity, nutritional improvement and decreased emotional eating).

3. To explore psychological changes related to participation in motivational interviewing treatment, including the adolescent's self-report of their health related quality of life, level of depression symptoms and self-esteem.

4. To compare physiological and anthropometric outcomes before and after psychological intervention.

#### Primary Hypotheses

**Figure 1:**
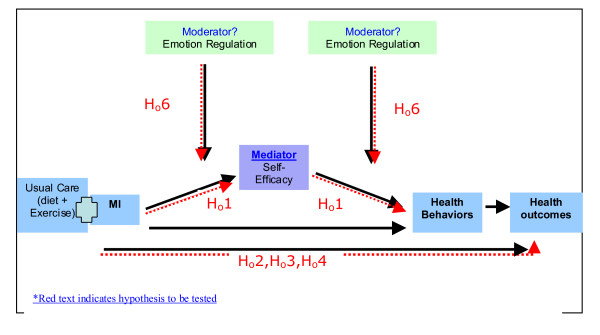
**'Theoretical Model of Hypotheses to be Tested'**.

**H**_**o**_**1**: At conclusion of the intervention (6 months) and 12 month follow-ups, in comparison to the control group, the MI group will demonstrate improved self-efficacy, as indicated on the Child Dietary Self-efficacy Scale [30] and the Weight Efficacy Lifestyle questionnaire [31]. ***(See ***Figure 1*** for a model of hypotheses to be tested)*.**

**H**_**o **_**2**: At conclusion of the intervention (6 months) and 12 month follow-ups, in comparison to the control group, the MI Group will report higher levels of healthy behaviors (i.e., increased activity as measured by the Weight Loss Behavior Scale [32] and the Dutch Eating Behavior Questionnaire [33]).

**H**_**o **_**3**: At conclusion of the intervention (6 months) and 12 month follow-ups, in comparison to the control group, the MI group will demonstrate increased improvement in psychological functioning as measured by the Peds Quality of Life questionnaire [34], the Child Depression Inventory [35] and the Rosenberg Self-Esteem Scale [36].

**H**_**o **_**4**: At conclusion of the intervention (6 months) and 12 month follow-ups, in comparison to the control group, the MI group will demonstrate improved physiological functioning/decreased metabolic risk as indicated by changes in BMI, body composition, improved lipid profile, insulin resistance, blood pressure and fitness;

**H**_**o **_**5**: Improvements in behavior change and self-efficacy will correlate with reduction in BMI and improved metabolic status.

#### Exploratory

**H**_**o **_**6**: Emotional self-regulation (as measured by the emotion scale on the Difficulties in Emotion Regulation Scale [37] and the Dutch Eating Behavior questionnaire [33]) will moderate the relationship between MI and behavior change.

#### Sample Size and Outcomes (Table 2)

The primary outcome for this study will be the total self-efficacy score as measured in the Child Dietary Self-Efficacy Scale (CDSS) [30]. Previous studies [38] have shown that the mean CDSS total self-efficacy score at in this population (without any intervention) varies between 5.0 and 6.5 with a standard deviation between 0.5 and 1.4. Previous behaviour studies in this population have shown treatment change in CDSS total self-efficacy scores between 1.1 and 1.9. Based on these preliminary data, assuming no differences between groups at baseline and a standard deviation of 1.0 (no differences between groups) we estimates that a sample size of 16 patients per group would allow the detection of between-group differences of 1.5 units with a standard deviation of 1.0 at an alpha level of 0.05 and 80% power. This estimate needs to be inflated to 20 patients per group in order to account for a 20% attrition rate.

**Table 2:** Analysis of Power

	EF = 1.25	EF = 1.5	EF = 1.75
Power			
80%	22	16	10
85%	26	18	12
90%	28	20	14

With this sample size we would be able to detect a difference of 1.4% in BMI percentile between groups over time assuming a 1.5% standard deviation, a difference of 2.85 points in self-esteem as measured in the Rosenberg scale (assuming a 3.15 points standard deviation and a difference of 6.5 points in the CDI depression scale (assuming a 7.1 points standard deviation). In linear regression models using maximum likelihood algorithm for parameter estimation (4 variables per observation in treatment group), this sample size would allow adjusting for 3 independent variables in addition to treatment group.

### Randomization

Once informed consent from subjects is obtained, participants will be randomly assigned to control group or treatment group based on a computer generated block randomization strategy stratified for age (10-14 and 14.1-18), and time of entry into the program (new vs. current participants). Subjects will have baseline measurements taken before randomization. At this time, a research assistant without direct contact with newly recruited participants will assign subjects to treatment or control group, based on a block randomization pattern stratified for age and program status.

**Figure 2:**
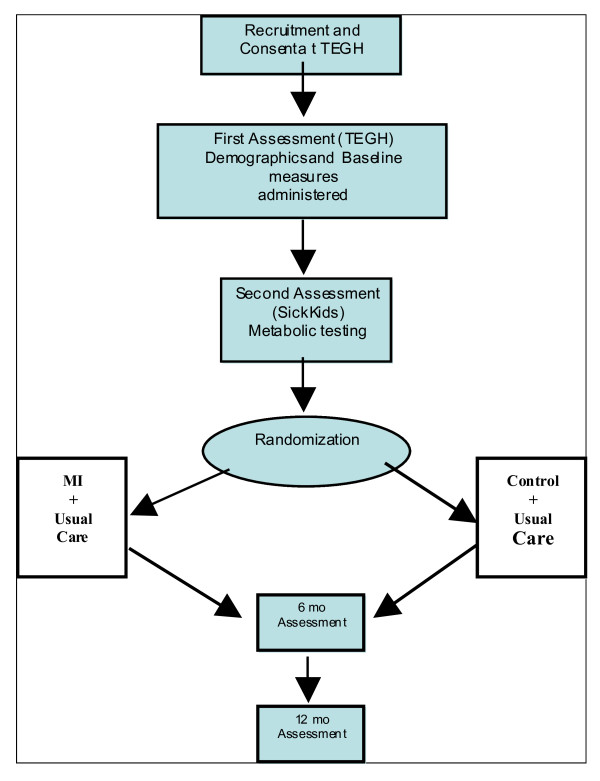
**'Flow Diagram of Recruitment and Study Procedure'**.

Participants are unaware of the specific psychological intervention, other than "we are testing two different types of psychological support." Study and program facilitators will partially aid in the administration of some of the measures, including BMI calculation. Both the experimental and control group will be blind to which group they were randomly assigned to. Baseline measures will be administered prior to beginning therapy intervention ***(See ***Figure 2*** for a flow diagram of recruitment protocol and study procedure)*.**

### Statistical Analysis

Analyses will be conducted with the Statistical Analysis System (SAS). Data will be described as means with standard deviation, median with interquartile range or frequencies as appropriate. A test of randomization (unpaired Student's t-test and Fisher's exact test) will be performed to ensure that all baseline variables are equally distributed between groups.

#### Primary Analyses

The mean changes in the primary outcome, self-reported self-efficacy scores, will be conducted for each group to determine possible between-group differences. Subsequent paired samples t-tests examining treatment vs. control group differences on secondary outcomes such as weight percentile, self esteem, quality of life and depression index will also be conducted. Repeated-measures ANOVA tests will be performed to assess changes in the aforementioned outcome variables over the study period.

#### Exploratory Analyses

A Pearson correlation coefficient will be used for univariate analysis. Change in self-efficacy will be correlated with psychological and physiological measures. The variables that are found to be the most significant will be entered into a linear regression model to determine what accounts for the variance in self-efficacy. Given the relatively small number of subjects, a maximum of 4 variables will be included in the regression analysis.

Analyses will also be conducted to explore the possible presence of a moderator; emotional self-regulation. A hierarchical multiple regression equation using SAS will be used to test for potential moderating effects.

#### Coding the MI

Therapy sessions will be taped and recordings will be coded using the Motivational Interviewing Treatment Integrity Coding System (MITI), version 3.1. A team of coders led by a trainer from the Motivational Interviewing Network of Trainers (MINT) will code the audio tapes. More than one coder will code each tape to ensure reliability of the coding. This will be important to help counter any bias that may have emerged during data interpretation.

## Discussion

Results from this study are hoped to enhance our understanding of the efficacy of MI with children and adolescents who are overweight or obese. If findings suggest that MI effects positive change to the treatment group over and above the control group intervention, and this correlates to improvement in psychological and physiological health, MI may have wider clinical applications. For example, the results of this study may generalize to other overweight and obese children and adolescents. External validity may be limited by participant demographics such as ethnicity, baseline obesity severity or comorbid diagnoses of weight related disorders such as diabetes, such that the results may not generalize to participants with characteristics that were not well represented in the study. Although attempts will be made to recruit participants of all ethnicities and participants will be matched for age and gender, certain types of participants may be underrepresented. Similarly, the results may not generalize beyond adolescents who are willing to volunteer for research or were motivated by the incentive of weight loss offered for participation. Another possible imitation to our study is the exclusion of parents. Future studies could include MI intervention with youth and their parents.

Health-related problems that accompany obesity are of great concern in the obese pediatric and adolescent population. Youth who are overweight have two to four times increased odds of having low scores for psychological health, self-esteem, and physical functioning [39]. Taken together, it is important that researchers develop empirical psychological interventions, grounded in theory, that will extend obesity treatment from support and education to promote sustained behavior change. As such, if significant results are found, Motivational Interviewing may be acknowledged as an effective treatment modality for child and adolescent obesity.

## Conflict of Interest

The authors declare that they have no competing interests.

## Authors' Contributions

BW contributed to the design and implementation of the study and is the author of this paper.  ED has contributed to the conceptualization and design of this study. BM contributed to the conceptualization and theory of this study. BMc is the nominated PI for the HISTORY team grant that has supported this study. JH has contributed to the overall design and coordination of the study. All Authors have read and approved the final manuscript.

## Authors' Information

BW is a PhD student in the clinical child psychology, developmental emphasis program at the University of Guelph. She is currently working on her doctoral dissertation in the area of childhood obesity interventions and is developing her skills as a clinician. ED is a psychologist and researcher at the Hospital for Sick Children. Her clinical and research activities have focused primarily on health psychology. She has played a part in many large research grants investigating health psychology issues. BM is a child psychologist with background in child health psychology and developmental psychology. BMc is the current director of the Cardiac Data Centre, the Paediatric Lipid Disorders Clinic and the Cardiovascular Clinical Research Unit at the Hospital for Sick Children.  JH is a pediatric endocrinologist, and co-principal investigator of the HISTORY study. She has expertise in conducting the physiologic tests of metabolic risk, and has experience conducting clinical trials in obese children and adolescents.

## Appendix

Subjects participating in the MI study will undergo a number of metabolic tests twice marking the start and end of the study. This testing will be carried out at the SickKids Hospital in the clinical investigation unit (CIU) and the exercise laboratory. Medical history will be obtained, including history of diabetes, hyperlipidemia, fatty liver disease, hypertension in participants and first-degree relatives.

A standard, calibrated scale and wall-mounted stadiometer will be used to measure weight and height, and BMI calculated as weight (kg)/height (m)^2^. Obesity is defined as BMI > 95^th^ percentile for age and gender on the Centre for Disease Control growth curves. Waist circumference will be measured in duplicate to the nearest 0.1 cm at the level of the umbilicus using a non-elastic fiberglass measuring tape (Tech-Med model 4414; Moore Medical Corp., New Britain, Conn). Three trails of above measurements will be completed and the mean taken for analyses. Systolic and diastolic blood pressure measurements will be taken using a Dinamapp device. The mean of 3 readings taken 1 minute apart in the right arm in seated position is recorded. Puberty will be assessed using the method of Tanner [40].

Following insertion of an intravenous catheter in the antecubital fossa, a fasting sample of blood is taken for measurement of glucose, insulin, triglyceride, low density lipoprotein (LDL) and high density lipoprotein (HDL) cholesterol, hepatic enzymes (ALT, AST), and highly sensitive C-reactive protein. Subjects are then given a glucose containing drink (75g Glucodex bottle: 1.75 g/kg to a max of 75 kg) and venous blood samples for insulin and glucose are obtained at 0, 30, 60, 90 and 120 minutes to screen for impaired glucose tolerance and type 2 diabetes. Insulin sensitivity (Si) is calculated by the Matsuda Model where; Si = 10 000/[(fasting glucose × fasting insulin) × (mean glucose × mean insulin)]^1/2^ where glucose is measured in mg/dl and insulin in μU/ml. This is well validated against gold-standard measures of insulin sensitivity in adults and in children with (r = 0.67 - 0.78) [41].

Physical activity will be assessed using the Habitual Activity Estimation Scale (HAES). Physical fitness will be measured using a maximal, incremental cycle ergometer (stationary bicycle) test, according to the Godfrey protocol. Similar to previous investigations, increments will be chosen based on the client's sex, height and physical activity level, so that the client terminates the test in 8-10 minutes due to exhaustion. Peak work capacity (VO_2_max) will be determined and compared to established norms. Additional exercise parameters include the degree of oxygen saturation with exercise, peak heart rate, peak ventilation, peak oxygen consumption and respiratory reserve (ratio of peak ventilation to maximum voluntary ventilation) [43,44].

## Pre-publication history

The pre-publication history for this paper can be accessed here:

http://www.biomedcentral.com/1471-2458/11/459/prepub
